# Dose–exposure–IGF-I response of once-weekly somapacitan in adults with GH deficiency

**DOI:** 10.1530/EJE-21-1167

**Published:** 2022-04-13

**Authors:** Rasmus Juul Kildemoes, Christian Hollensen, Beverly M K Biller, Gudmundur Johannsson, Yutaka Takahashi, Michael Højby Rasmussen

**Affiliations:** 1 Global Development, Novo Nordisk A/S, Bagsvaerd, Denmark; 2 Neuroendocrine and Pituitary Tumor Clinical Center, Massachusetts General Hospital, Boston, Massachusetts, USA; 3 Institute of Medicine, Sahlgrenska Academy, University of Göteborg, Göteborg, Sweden; 4 Department of Endocrinology, Sahlgrenska University Hospital, Göteborg, Sweden; 5 Department of Diabetes and Endocrinology, Nara Medical University, Kashihara, Japan; 6 Division of Diabetes and Endocrinology, Department of Internal Medicine, Kobe University Graduate School of Medicine, Kobe, Japan; 7 Clinical Drug Development, Novo Nordisk A/S, Søborg, Denmark

## Abstract

**Objective:**

Growth hormone (GH) replacement therapy in patients with adult growth hormone deficiency (AGHD) is individually titrated due to variable dose–responses among patients. The aim of this study was to provide clinical guidance on dosing and titration of the novel long-acting GH derivative somapacitan based on analyses of somapacitan dose–insulin-like growth factor I (IGF-I) responses in AGHD patients.

**Design:**

Analyses of dosing information, 4364 somapacitan concentration samples and 4880 IGF-I samples from 330 AGHD patients treated with somapacitan in three phase 3 trials.

**Methods:**

Pharmacokinetic/pharmacodynamic modelling was used to evaluate starting dose groups by age and oral oestrogen therapy, characterise the dose–IGF-I response in the overall AGHD population and patient subgroups, predict the IGF-I response to dose changes and simulate missed dosing.

**Results:**

The analyses supported the clinical recommendations of higher starting doses for younger patients and women on oral oestrogen replacement therapy. For patients switching from daily GH treatment, the mean maintenance dose ratio between somapacitan (mg/week) and somatropin (mg/day) was predicted to be 8.2 (observed interquartile range of 6.7–9.1). Simulations of IGF-I SDS profiles confirmed the appropriate time for IGF-I sampling to be 3–4 days after somapacitan dosing and supported somapacitan administration with up to 3 days delay in case of missed dosing. Subgroup analyses characterised the dose–exposure–IGF-I response in patient subgroups and indicated that dose requirements are mainly influenced by sex and oral oestrogen treatment.

**Conclusions:**

This study extends the knowledge of the somapacitan dose–IGF-I response and provides information on clinical dosing of once-weekly somapacitan in patients with AGHD.

## Introduction

Patients with adult growth hormone deficiency (AGHD) require growth hormone (GH) replacement therapy to restore GH-mediated effects on metabolism and body composition and to prevent long-term complications of AGHD (1, 2). Dose requirements for GH replacement vary according to a number of clinical factors, including age, sex and interaction with other hormone replacement therapies (particularly oral oestrogen), which contribute to variable dose–responses among patients (3, 4). Due to these variations, GH treatment in adults is initiated at low starting doses and individually titrated based on clinical response, adverse reactions and serum insulin-like growth factor I (IGF-I) levels (5, 6).

IGF-I is currently the most widely accepted biomarker for titration and monitoring of GH replacement in AGHD (7). Serum IGF-I levels are monitored during dose titration and after the GH maintenance dose has been achieved, with the aim of reaching IGF-I levels observed in the healthy population. Clinical guidelines recommend that the titration of GH treatment is performed to levels in the normal range, as indicated by an IGF-I standard deviation score (SDS) of -2 to +2 (6), or to levels in the upper normal range of 0 to +2 (5).

Current GH replacement therapy is administered as daily s.c. injections. Daily treatment regimens can be burdensome to patients with AGHD, who often require long-term or lifelong treatment. Indeed, several studies have reported poor treatment adherence and persistence in patients with AGHD (8, 9, 10, 11). Long-acting GH (LAGH) formulations with decreased injection frequency may reduce the burden of treatment for AGHD patients, thereby potentially improving adherence and clinical outcomes.

Somapacitan is a novel, reversible albumin-binding GH derivative with a single substitution in the GH amino acid backbone, to which an albumin-binding moiety is attached (12). The non-covalent reversible association to albumin delays the elimination of somapacitan after s.c. injection, thereby prolonging the *in vivo* half-life, making once-weekly administration possible.

The different pharmacokinetic (PK) and pharmacodynamic (PD) properties of LAGH formulations, compared with daily GH products, prompt a need for clinical guidance on their use. In the present study, we used PK/PD modelling based on data from 330 patients to provide supportive data on the use of somapacitan in patients with AGHD. The analyses evaluated starting doses, dose titration and therapeutic dose ranges. In addition, modelling analyses were used to assess dose requirements in patients switching from daily GH, provide insights on appropriate IGF-I sampling times, support missed dosing guidelines and characterise the dose–exposure–IGF-I response in patient subgroups.

## Subjects and methods

### Trials providing data

Somapacitan PK and PK/PD models were based on data from patients with AGHD randomised to somapacitan (Sogroya^®^, Novo Nordisk)) in three placebo- or active-controlled (somatropin (Norditropin^®^ NordiFlex^®^, Novo Nordisk)) phase 3 trials (ClinicalTrials.gov identifiers: NCT02229851 (REAL 1), NCT02382939 (REAL 2), NCT03075644 (REAL JP) (13, 14, 15)) (Table 1).

**Table 1:** Overview of trials and patients.

	REAL 1	REAL 2	REAL JP
Study design	Phase 3, randomised, placebo-controlled (double-blind) and active-controlled (open-labelled)	Phase 3, randomised, active-controlled, open-labelled	Phase 3, randomised, active-controlled, open-labelled
Treatment duration	34 weeks (main)^a^ + 52 weeks (extension)^a^	26 weeks	52 weeks
Trial duration	88 weeks (34 weeks treatment, 1 week wash-out, 52 weeks treatment, 1 week wash-out)	27 weeks (26 weeks treatment, 1 week wash-out)	53 weeks (52 weeks treatment, 1 week wash-out)
Patients enrolled	Patients with AGHD	Patients with AGHD	Patients with AGHD
	Treatment-naïve	Previously treated	Previously treated
Population	Global	Global	Japanese
Comparator	Placebo; somatropin	Somatropin	Somatropin
Patients randomised	301	92	62
Patients treated with somapacitan	120 (main)^a^	61	46
	220 (extension)^a^		
	226 unique patients		
Patients treated with somatropin	119 (main)^a^	31	16
	52 (extension)^a^		

^a^REAL 1 comprised a main trial period followed by an extension trial period. In the main trial period, patients were randomised to somapacitan, placebo or somatropin at the ratio of 2:1:2, respectively. In the extension trial period, placebo-treated patients were re-allocated to somapacitan and patients randomised to somatropin were re-allocated to somapacitan or somatropin at the ratio of 1:1, respectively.

AGHD, adult growth hormone deficiency.

REAL 1 and REAL 2 were global trials, and REAL JP was conducted in Japan. REAL 1 enrolled GH treatment-naïve patients and comprised a main trial period and an extension trial period, separated by a 1-week washout. Data from both trial periods were included in modelling. REAL 2 and REAL JP enrolled patients were previously treated with human growth hormone (hGH). Further details on the trials, including eligibility criteria, were published previously (13, 14, 15).

Each trial was approved by the relevant local and national ethics committees and was conducted in accordance with the International Conference on Harmonisation guidelines for Good Clinical Practice (16) and the Declaration of Helsinki (17). Informed consent was obtained from all patients prior to inclusion. An overview of ethics committees for each trial is included in Supplementary Table 1 (see section on supplementary materials given at the end of this article).

### Dosing regimen and dose titration

Somapacitan was administered once-weekly as s.c. injections and somatropin as once-daily s.c. injections. Patients were assigned to one of three starting dose levels by age group (patients ≤60 years old: 1.5 mg/week; patients >60 years old: 1.0 mg/week) or oral oestrogen replacement therapy (females on oral oestrogen : 2.0 mg/week), based on dose recommendations for daily GH treatment in patients with AGHD (18).

Dose titrations were performed in pre-defined time periods. In REAL 1 (treatment-naïve patients), somapacitan doses were titrated every second week over 8 weeks, according to IGF-I SDS levels and IGF-I SDS change from screening. In the extension period, patients treated with daily somatropin in the main period were re-randomised to daily somatropin or once-weekly somapacitan (1:1). Identical dose titration regimens from starting dose levels were applied in the main and extension trial periods. In REAL 2 and REAL JP (previously treated patients), dose adjustments were performed over a period of 8 weeks and 20 weeks, respectively, and were based on IGF-I SDS levels.

Titration algorithms specified the IGF-I SDS target range (from −0.5 to 1.75 in REAL 1 and from 0 to 2 in REAL 2 and REAL JP) and the recommended somapacitan dose adjustment based on the observed IGF-I SDS levels.

After dose titration, individual dose levels were fixed for the remaining duration of the trials. The somapacitan dose range was 0.1–8 mg/week, and the somatropin dose range was 0.05–1.1 mg/day. Dosing information including actual dosing time and dose level was available from patient-reported dosing diaries.

### Blood sampling and bioanalysis

Serial blood sampling was performed throughout the phase 3 trials at carefully selected time-points relative to dosing, in order to cover baseline (for treatment-naïve patients), maximum and trough levels of PK and IGF-I following somapacitan dosing. IGF-I sampling for somapacitan dose titration was performed on days 3–4 after dosing, as IGF-I measurements during this interval were expected to resemble weekly average levels (19). IGF-I SDS was calculated using the reference data published by Bidlingmaier *et al.* (20). Somapacitan and IGF-I assays are described in the Supplementary Methods.

### Somapacitan population PK and PK/PD analysis methods

Somapacitan population PK and PK/PD models in patients with AGHD were based on previously published models developed from full PK and IGF-I profiles obtained in phase 1 (21) and refitted to sparse PK and IGF-I data collected in phase 3. For comparison between somapacitan and daily GH treatment, a PK/PD model for somatropin was developed based on data from AGHD patients treated with somatropin in the same phase 3 trials (Tables 1 and 2). Details regarding data sets and data cleaning, model development, modelling analyses and software are included in the Supplementary Methods, Supplementary Fig. 1 and Supplementary Table 2.

**Table 2:** Demographics and characteristics of patients included in the analysis. Data are given as *n* (%), mean ± s.d. or range. Patients with at least one PK sample were included in the analyses.

Characteristics	REAL 1 (%)	REAL 2 (%)	REAL JP (%)	Total somapacitan (%)	Total somatropin (%)
All, *n*	225 (68.2)	59 (17.9)	46 (13.9)	330 (100)	164 (100)
Population					
hGH treatment-naïve	225 (100)	–	–	225 (68.2)	118 (72)
Previously hGH-treated	–	59 (100)	46 (100)	105 (31.8)	46 (28)
Gender					
Female	119 (52.9)	26 (44.1)	22 (47.8)	167 (50.6)	83 (50.6)
Male	106 (47.1)	33 (55.9)	24 (52.2)	163 (49.4)	81 (49.4)
Race^a^					
White/other	161 (71.6)	47 (79.7)	–	208 (63)	106 (64.6)
Asian Japanese	36 (16)	11 (18.6)	46 (100)	93 (28.2)	40 (24.4)
Asian non-Japanese	28 (12.4)	1 (1.7)	–	29 (8.8)	18 (11)
Ethnicity					
Not Hispanic or Latino/unknown	210 (93.3)	59 (100)	46 (100)	315 (95.5)	156 (95.1)
Hispanic or Latino	15 (6.7)	–	–	15 (4.5)	8 (4.9)
Age group					
18–64 years	196 (87.1)	48 (81.4)	34 (73.9)	278 (84.2)	136 (82.9)
From 65 years	29 (12.9)	11 (18.6)	12 (26.1)	52 (15.8)	28 (17.1)
Body weight (kg)					
Mean ± s.d.	74.2 ± 21.7	82.3 ± 17.9	69.4 ± 22.7	75 ± 21.5	76.1 ± 21.9
Range	36–140.9	46.8–121.8	34.5–150.5	34.5–150.5	22.5–151.2
Age (years)					
Mean ± S.D.	44.6 ± 14.8	48 ± 16.4	54.1 ± 12.1	46.5 ± 15.1	47.3 ± 15.4
Range	23–75	19–77	20.2–75.2	19–77	20–77
Start dose group					
Patients > 60 years	38 (16.9)	16 (27.1)	16 (34.8)	70 (21.2)	42 (25.6)
Patients ≤ 60 years	128 (56.9)	34 (57.6)	26 (56.5)	188 (57)	96 (58.5)
Females on oral oestrogen	59 (26.2)	9 (15.3)	4 (8.7)	72 (21.8)	26 (15.9)

^a^Race groups with fewer than 20 patients were included in the White/other group.

hGH, human growth hormone.

## Results

### Data for analyses

Somapacitan analyses were based on data from 330 patients with AGHD randomised to somapacitan in three placebo- or active-controlled (somatropin) phase 3 trials (Table 2). Somatropin analyses were based on data from AGHD patients treated with daily somatropin in the same trials. Baseline demographics and characteristics are shown in Table 2. Demographics and characteristics for the starting dose groups are included in Supplementary Table 3.

**Figure 1:**
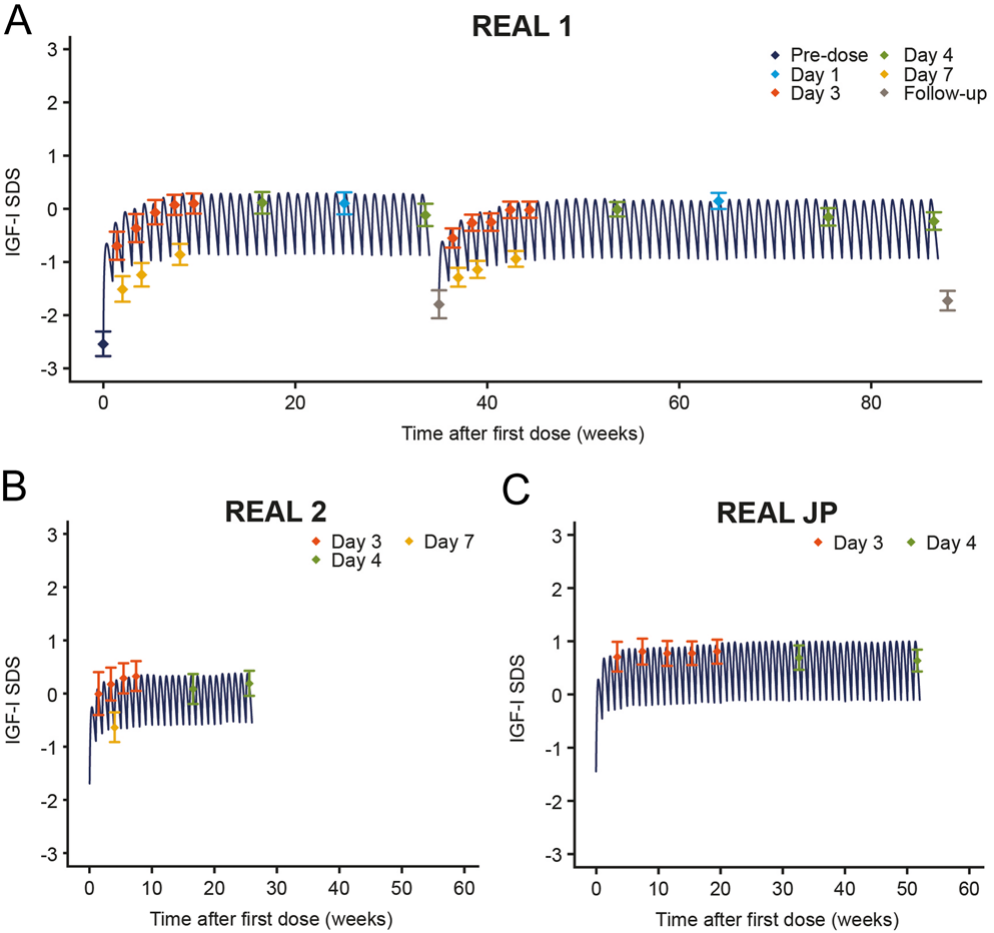
IGF-I sampling schedule for REAL 1 (A), REAL 2 (B) and REAL JP (C). Lines are means of individual model predictions obtained with the final PK/PD model. Symbols with error bars are mean IGF-I levels with 95% CIs and indicate sampling times. Symbols are coloured by sampling day after the latest dose. IGF-I, insulin-like growth factor I; PD, pharmacodynamic; PK, pharmacokinetic; SDS, standard deviation score. A full colour version of this figure is available at https://doi.org/10.1530/EJE-21-1167.

Following data cleaning, a total of 4364 PK concentration values and 4880 IGF-I values from 330 patients were included in the final somapacitan PK and PK/PD data sets, respectively. Details on data cleaning are included in the Supplementary Methods. The collected data supported the fitting of the population PK and PK/PD models, including characterisation of baseline (for treatment-naïve patients), maximum and trough levels of PK and IGF-I (Fig. 1).

### Starting doses and dose titration in AGHD patients

The patients were divided in three starting dose groups based on age group or oral oestrogen replacement therapy (Table 3). In accordance with the starting dose groups, observed doses after titration were highest for females on oral oestrogen and lowest for patients > 60 years (Table 3). Starting dose levels were relatively low for all groups, as indicated by the higher doses after titration. The majority of patients increased or maintained the starting dose, and most patients requiring titration reached maintenance dose level within 1–2 titration visits (Supplementary Table 4).

**Table 3:** Somapacitan starting doses and doses after titration. Starting doses used in phase 3 trials and mean maintenance doses after titration across phase 3 trial.

	Somapacitan		Somatropin	
	Starting doses	Fixed doses	Starting doses	Fixed doses
Patients ≤ 60 years old	1.50 mg/week	2.1 mg/week	0.20 mg/day	0.28 mg/day
Patients > 60 years old	1.00 mg/week	1.4 mg/week	0.10 mg/day	0.17 mg/day
Females on oral oestrogen	2.00 mg/week	3.8 mg/week	0.30 mg/day	0.51 mg/day
All patients	1.50 mg/week	2.4 mg/week	0.19 mg/day	0.29 mg/day

**Figure 2:**
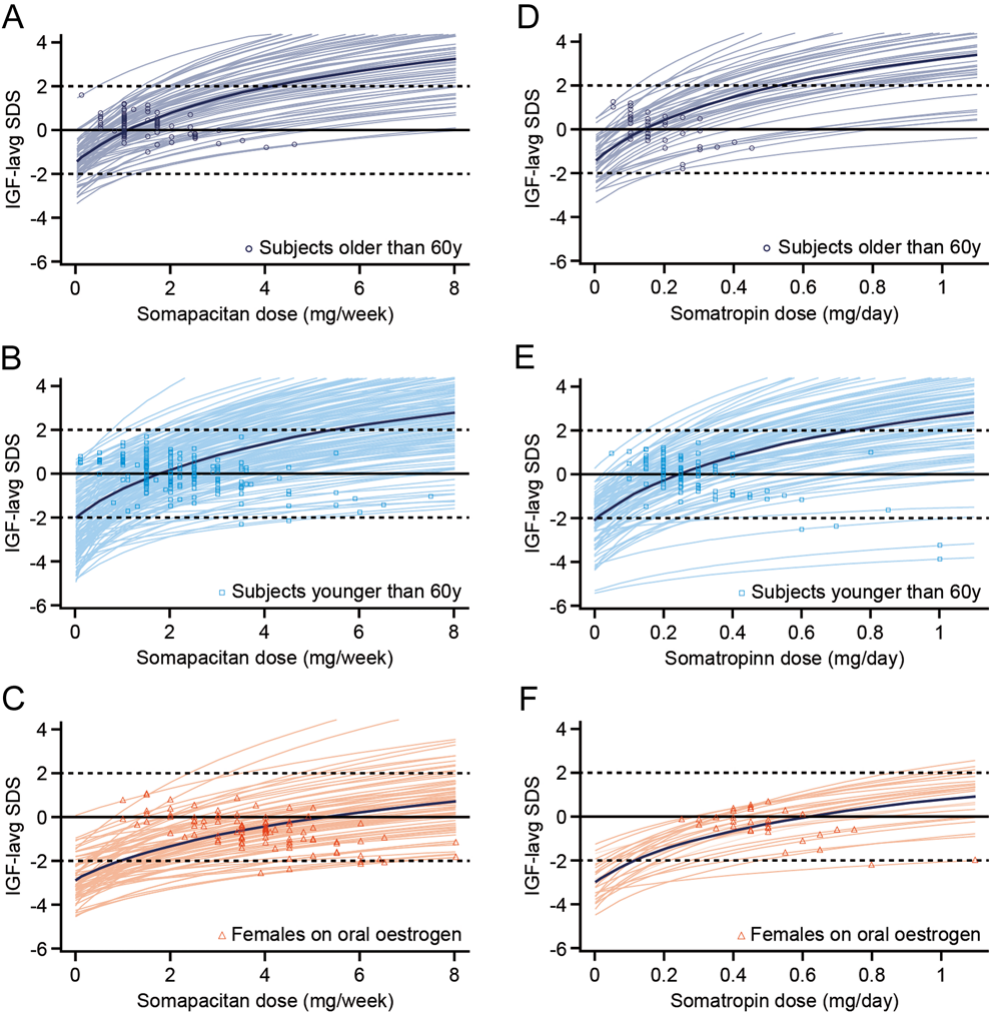
Observed and predicted dose–IGF-I responses for somapacitan and somatropin by starting dose group. Open symbols (circles, squares and triangles) are observed dose–IGF-I response pairs at the phase 3 maintenance dose levels for somapacitan (A, B and C) or somatropin (D, E and F). Lines are individual (thin lines) and mean (thick lines) predicted dose–response relationships across the somapacitan (0.1–8 mg/week) or somatropin (0.05–1.1 mg/day) dose ranges. Intersects for starting dose groups; patients > 60 years: 0 SDS, somapacitan 1.1 mg/week and 2 SDS, 4.2 mg/week; patients ≤ 60 years: 0 SDS, 1.8 mg/week and 2 SDS, 5.5 mg/week; Females on oral oestrogen: 0 SDS, 5.5 mg/week and 2 SDS, above 8 mg/week. IGF-I_avg_ SDS, average insulin-like growth factor I during maintenance treatment standard deviation score. A full colour version of this figure is available at https://doi.org/10.1530/EJE-21-1167.

To further evaluate the expected therapeutic dose range, modelling analyses were used to predict the dose–IGF-I response for each individual patient in phase 3 by starting dose group across the somapacitan (0.1–8 mg/week) or somatropin (0.05–1.1 mg/day) dose ranges (Fig. 2). Patients > 60 years were on average predicted to reach the upper normal IGF-I SDS range (0 SDS) with a somapacitan dose of 1.1 mg/week and to exceed the upper normal range (2 SDS) at doses above 4.2 mg/week. Patients ≤ 60 years were on average predicted to reach 0 and 2 SDS with doses of 1.8 and 5.5 mg/week, respectively. Females on oral oestrogen therapy were on average predicted to require a dose of 5.5 mg/week to reach 0 SDS, while the mean predicted IGF-I response did not reach 2 SDS at the maximum dose of 8 mg/week, indicating that most patients in this group will not exceed the upper normal IGF-I SDS range at this dose level. On a patient level, the dose required to obtain a normal range IGF-I SDS varied across the interval of the dose range for both somapacitan and somatropin. The dose–IGF-I responses indicated that a subset of patients on oral oestrogen may have benefitted from further uptitration than allowed by the pre-specified titration schedule in phase 3 (Fig. 2C and F).

### Switching from daily GH to once-weekly somapacitan

The mean dose level after titration in phase 3 was 2.364 mg/week somapacitan and 0.289 mg/day somatropin (Table 3). This is equivalent to an effective dose ratio between somapacitan (mg/week) and somatropin (mg/day) of 8.2 for the population. Modelling confirmed that this effective ratio applies across the dose and IGF-I range (Supplementary Table 5).

The individual variation in effective dose ratio was investigated by comparing doses after titration in 48 subjects who switched from daily somatropin in the main phase of REAL 1 to once-weekly somapacitan in the extension phase. In this group, the ratio between somapacitan (mg/week) and somatropin (mg/day) was 8.2 with a range of 4.3–18 (interquartile range (IQR) of 6.7–9.1), when excluding two outliers.

**Figure 3:**
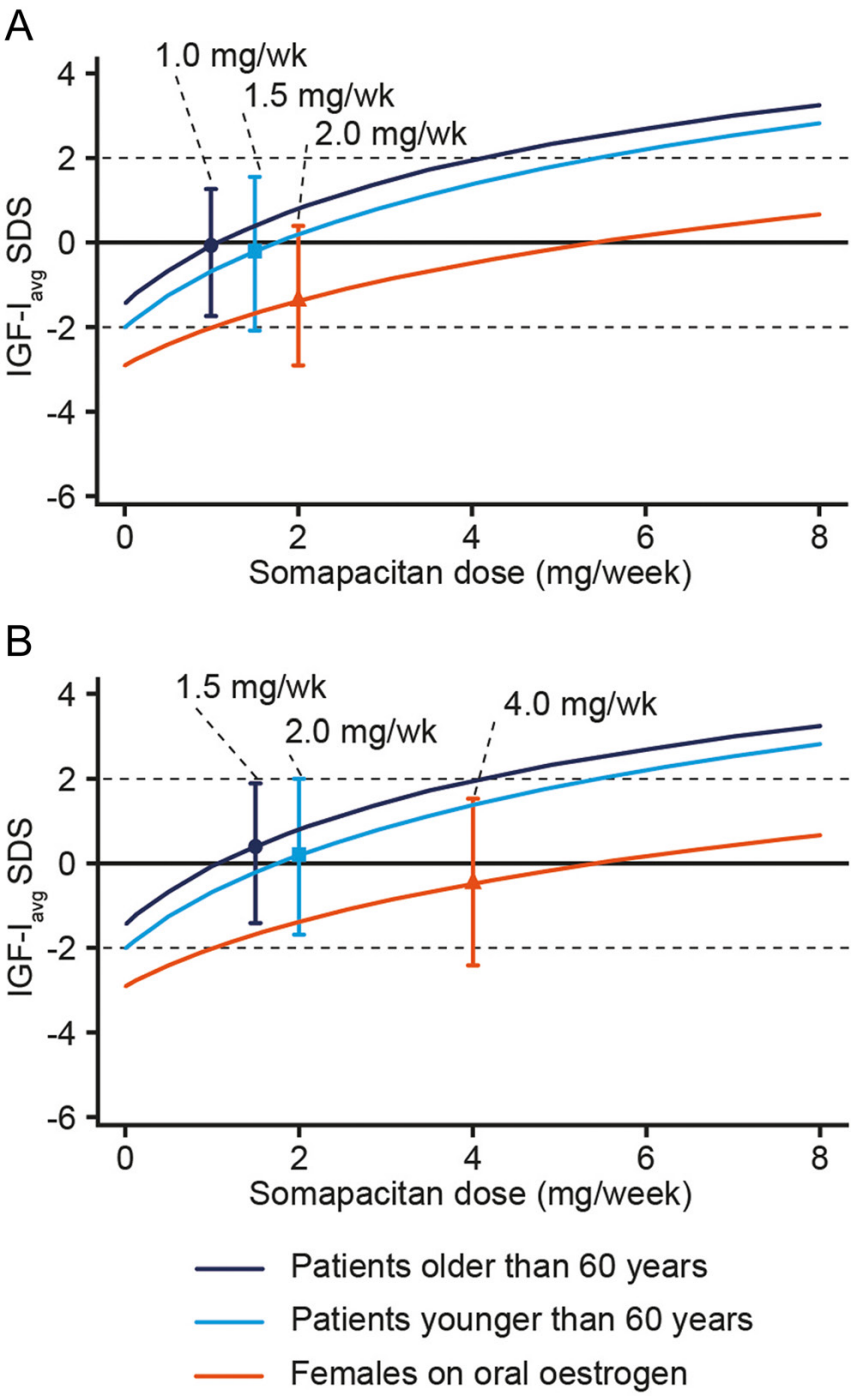
Prediction of IGF-I distribution for various starting doses. Lines are means of individual dose–response curves by starting dose group. Symbols and bars are means and 5th to 95th percentiles at starting dose levels of 1.0, 1.5 or 2.0 mg/week used in phase 3 (A) and at higher doses of 1.5, 2.0 or 4.0 mg/week (B), for patients younger than 60 years, patients older than 60 years and females on oral oestrogen, respectively. IGF-I_avg_ SDS, average insulin-like growth factor I during maintenance treatment standard deviation score; wk, week. A full colour version of this figure is available at https://doi.org/10.1530/EJE-21-1167.

Depending on the local regulations, patients switching from daily GH to somapacitan may either start somapacitan treatment on the same starting doses as treatment-naïve patients or on alternative higher starting doses, in order to reduce the time required to achieve the optimal therapeutic dose. Modelling analyses were used to compare expected IGF-I SDS levels at treatment initiation with low starting doses (1.0, 1.5 or 2.0 mg/week based on age and oral oestrogen replacement therapy) or higher starting doses (1.5, 2.0 or 4.0 mg/week) (Fig. 3). The low doses were predicted to result in a mean IGF-I SDS of −0.4, with approximately 1% of patients having IGF-I SDS > 2 and 10% with IGF-I SDS < –2 (Fig. 3A), whereas the higher starting doses were predicted to result in a mean IGF-I SDS of 0.1, with approximately 5% of patients having IGF-I SDS > 2 and 5% with IGF-I SDS < –2 (Fig. 3B).

### Weekly IGF-I SDS profile and monitoring of IGF-I

**Figure 4:**
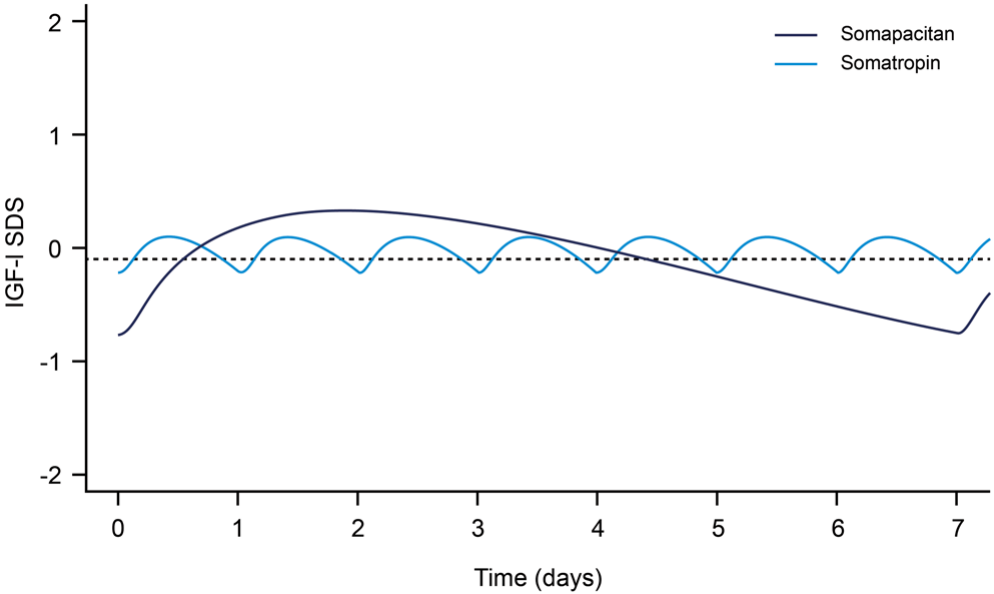
Weekly IGF-I SDS profiles following somapacitan and somatropin dosing. Full lines are means of individual predictions in the fixed dose periods after titration as observed in phase 3 for somapacitan (mean dose, 2.4 mg weekly) and somatropin (mean dose, 0.3 mg daily). The dashed line represents the weekly average IGF-I SDS for somapacitan (0.09 SDS). IGF-I, insulin-like growth factor I; SDS, standard deviation score. A full colour version of this figure is available at https://doi.org/10.1530/EJE-21-1167.

For a comparison of IGF-I profiles following somapacitan and somatropin dosing, weekly IGF-I SDS profiles were simulated based on the average maintenance dose levels observed in phase 3 (Fig. 4). The somapacitan IGF-I SDS profile reached its maximum approximately 2 days after dosing and differed from the profile of weekly somatropin. Average weekly IGF-I SDS levels for somapacitan and somatropin were overall similar at the phase 3 maintenance dose levels. Sampling for IGF-I monitoring is recommended on days 3–4 after dosing, as IGF-I SDS levels during this interval are expected to be similar to weekly average levels.

### Missed doses of somapacitan and daily somatropin

To assess the impact of delayed doses of somapacitan compared with missed doses of daily GH, modelling was used to simulate IGF-I SDS profiles for 1 or 3 days of delayed/missed dosing.

**Figure 5:**
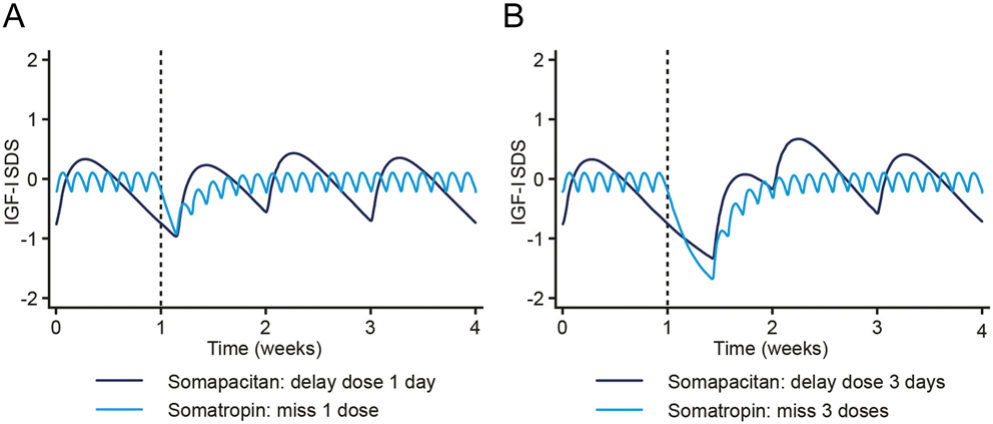
IGF-I SDS profiles following missed doses of somapacitan and somatropin. Lines are means of individual predictions in simulated scenarios with somapacitan dosing delayed or somatropin dosing missed for 1 (A) or 3 (B) days. Dotted vertical lines represent time of the first missed dose. IGF-I, insulin-like growth factor I; SDS, standard deviation score. A full colour version of this figure is available at https://doi.org/10.1530/EJE-21-1167.

After one missed dose of somatropin, 3–4 days of subsequent dosing were required to restore the regular maintenance treatment IGF-I profile; however, 1 day of delayed somapacitan dosing appeared to have minor impact on the IGF-I SDS profile (Fig. 5A). After 3 days of missed dosing, 4–5 days of somatropin dosing were needed to return to the maintenance treatment profile (Fig. 5B). In contrast, somapacitan was predicted to largely maintain IGF-I levels in accordance with the maintenance treatment profile, despite a 3-day delay of dosing. Three days of delayed/missed dosing were predicted to reduce IGF-I levels by on average 0.4 SDS for somatropin and 0.1 SDS for somapacitan, over the period of 2 weeks required to stabilise the IGF-I profiles starting from the planned dosing day.

### Dose–exposure–IGF-I response in patient subgroups

**Figure 6:**
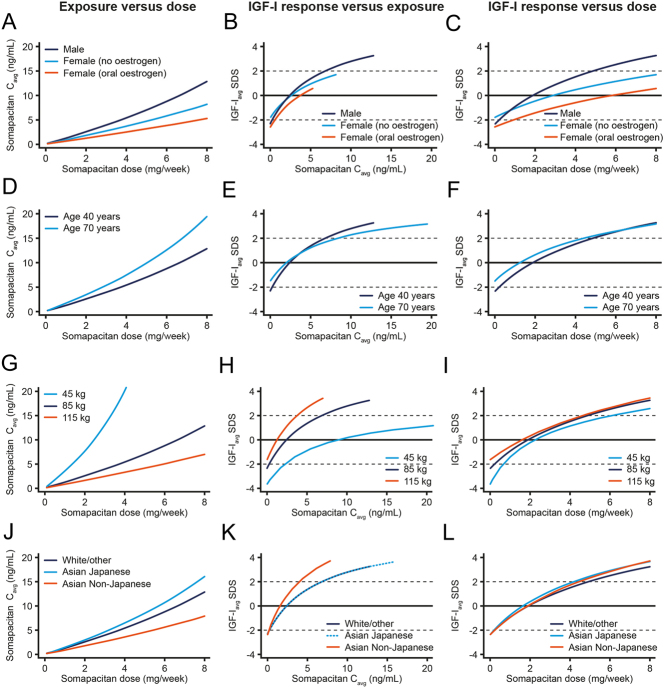
Somapacitan dose–exposure-IGF-I response in patient subgroups. Lines are dose–exposure, exposure–IGF-I response and dose–IGF-I response relationships for patient demographics and characteristics significantly impacting somapacitan PK and/or PD: Sex and oral oestrogen therapy (A-C), age (D-F), body weight (G-I) and race (J-L). The pre-specified reference subject was male, White, 85 kg, age 40 years. C_avg_, average somapacitan exposure during maintenance treatment; IGF-I_avg_ SDS, average insulin-like growth factor I during maintenance treatment standard deviation score; PD, pharmacodynamic; PK, pharmacokinetic. A full colour version of this figure is available at https://doi.org/10.1530/EJE-21-1167.

Sex and oral oestrogen therapy, age, body weight and race were identified as factors with statistically significant impact on somapacitan PK and PD (Supplementary Table 2). To provide scientific insights into the variable dose requirements across patient subgroups, the influence of these factors on the somapacitan dose–exposure–IGF-I response was characterised using modelling analyses (Fig. 6). The employed modelling approach allowed for a separate evaluation of each patient categorisation (sex and oral oestrogen treatment, age, body weight, race) by accounting for the influence of the other categories in each analysis.

Estimated dose–IGF-I responses indicated that mean IGF-I SDS was higher in males compared to females, and in particular, females on oral oestrogen replacement, at equivalent somapacitan doses (Fig. 6C). The lower dose–response in women and women on oral oestrogen was the combined result of lower serum drug concentrations per somapacitan dose level (Fig. 6A) and lower IGF-I responses at matching somapacitan concentrations (Fig. 6B), of which the dose–exposure effect was the most influential.

The impact of age is illustrated by estimated dose–responses for patients aged 40 (representing age group <65) and 70 years (representing age group ≥65 years). Somapacitan exposure was higher in patients ≥ 65 years across the somapacitan dose range (Fig. 6D). Baseline IGF-I SDS was lower in younger compared with older patients, but the IGF-I response to somapacitan was higher (Fig. 6E). The resulting dose–IGF-I responses indicated that mean IGF-I SDS is higher in patients ≥ 65 years compared with younger patients, at equivalent dose levels (Fig. 6F).

The influence of body weight is illustrated by estimated dose–responses for patients weighing 45, 85 (reference) and 115 kg. Somapacitan serum concentrations increased with decreasing body weight at equivalent dose levels (Fig. 6G); however, patients with low body weight needed higher serum somapacitan concentrations to obtain similar IGF-I responses as patients with higher body weight (Fig. 6H). As a result, dose–IGF-I responses were relatively similar across body weights (Fig. 6I).

Somapacitan dose–exposure and exposure-IGF-I responses varied across race groups (Fig. 6J and K), but the resulting dose–IGF-I responses were similar (Fig. 6L). Hence, different race groups needed equivalent somapacitan doses to reach similar IGF-I SDS levels.

## Discussion

In the present study, we analysed the dose–IGF-I response of patients with AGHD based on data from 330 AGHD patients enrolled in phase 3 of the somapacitan clinical development programme. The analyses supported the differentiation of somapacitan starting doses by age group (≤60 years; >60 years) and oral oestrogen replacement therapy. Dose–response predictions indicated that higher starting doses may be relevant for patients switching from daily GH treatment and predicted a mean maintenance dose ratio for once-weekly somapacitan (mg/week) and daily somatropin (mg/day) of 8.2 (IQR of 6.7–9.1). Simulations of IGF-I SDS profiles confirmed the appropriate time for IGF-I sampling to be 3–4 days after somapacitan dosing and supported somapacitan administration with up to 3 days delay in case of missed dosing. Analyses in patient subgroups characterised the relative dose requirements according to factors with significant impact on the somapacitan dose–exposure-IGF-I response (sex and oral oestrogen replacement, age, body weight and race) and indicated that dose requirements are mainly influenced by sex and oral oestrogen treatment. These results may support clinical guidance on the use of once-weekly somapacitan in patients with AGHD.

### Dose considerations for starting doses and dose titration

In accordance with general guidelines on GH treatment (18), patients in phase 3 were assigned to one of three starting dose groups by age or oral oestrogen replacement therapy, based on the expectation that a similar distribution in therapeutic dose levels would apply for somapacitan and daily GH treatment. The phase 3 starting dose groups were confirmed to be appropriate, as indicated by the differences in observed dose levels after titration as well as model-based dose–response analyses.

The majority of patients in phase 3 reached the maintenance dose level after 1–2 titration visits with titration steps of 0.5–1.5 mg/week. The treatment target in phase 3 was set as the upper normal IGF-I SDS range (−0.5 to 1.75 SDS in REAL 1 and 0 to 2 SDS in REAL 2 and REAL JP), in accordance with GH treatment guidelines (18). Dose–IGF-I response predictions showed that the upper normal IGF-I range (0–2 SDS) for each patient is covered by a relatively large dose range, indicating that there is room for optimising dosing to clinical response within the upper normal IGF-I range for most patients. While IGF-I serves to guide titration of GH replacement therapy, it should, however, be emphasised that the IGF-I SDS level needed to achieve a clinically relevant response is individual and that IGF-I levels do not strongly correlate with clinical endpoints (7). Indeed, patients may experience satisfactory treatment effects at IGF-I SDS levels below 0 SDS, as the effect also depend on the obtained IGF-I SDS change from baseline, the individual clinical response, as well as adverse effects.

### Dose considerations when switching from daily GH treatment to somapacitan

The somapacitan maintenance dose (mg/week) for patients switching from daily GH treatment is on average expected to be 8.2 times higher than the previous somatropin maintenance dose (mg/day). This dose ratio may be informative for setting expectations for maintenance dose levels of somapacitan following the switch from daily GH treatment. The effective ratio for each patient is, however, individual and depends on the patient’s dose–response for each compound. This is illustrated by the range of effective dose ratios (IQR of 6.7–9.1; range of 4.3–18 for somapacitan (mg/week) and somatropin (mg/day)) observed in the patients who switched from daily somatropin to once-weekly somapacitan in REAL 1. The mean population ratio therefore does not substitute the requirement for individual titration to identify the optimal somapacitan dose for all patients with AGHD. As the approved somapacitan posology varies across regions for patients switching from daily GH to once-weekly somapacitan, these patients may receive higher starting doses than treatment-naïve patients, if in accordance with local regulations.

### IGF-I monitoring during somapacitan treatment

Sampling for IGF-I measurements during dose titration or maintenance treatment for a LAGH product requires knowledge of the weekly IGF-I profile, as it varies over the dosing interval. In clinical practice, as in the phase 3 trials, IGF-I sampling for somapacitan titration should be performed on days 3–4 after dosing. IGF-I measurements taken during this interval resemble weekly average IGF-I levels (19). Sampling for IGF-I should be performed when the IGF-I profile has reached steady state, which occurs after 1–2 weekly somapacitan doses. Following dose adjustments, the somapacitan IGF-I profile is expected to adapt to the new steady-state level within the same duration of 1–2 dosing intervals.

### Missed dosing

Considering the challenges with treatment adherence and persistence reported for patients with AGHD (8, 9, 10), the impact of missed doses on GH-induced responses is considered highly relevant. Simulations of missed somapacitan or somatropin doses indicated that IGF-I levels were maintained despite 1–3 days of delayed somapacitan dosing compared with 1–3 missed doses of daily somatropin. Accordingly, the prescribing information of somapacitan allows the patient to administer treatment up to 3 days after the planned dose. This offers patients a window to administer treatment if they forget a dose on a specific day, as opposed to missing up to three doses of daily GH. The opportunity for delayed dosing is an improvement in treatment flexibility for patients with AGHD, in addition to the reduced injection frequency (from 365 to 52 injections per year), when switching from daily hGH to once-weekly somapacitan. Reductions in treatment frequency have previously been reported to increase treatment adherence in other chronic diseases, such as major depressive disorder, osteoporosis and attention-deficit hyperactivity disorder (22, 23, 24).

### Dose–exposure–IGF-I response in patient subgroups

Dose requirements for GH replacement therapy in patients with AGHD vary according to individual patient demographics and characteristics (4). This was reflected in the variable somapacitan dose levels required to obtain normal serum IGF-I SDS levels in the phase 3 population, emphasising the need for individualised dose titration across patient subgroups.

To provide insights on the patient demographics and characteristics influencing somapacitan dose requirements, dose–exposure–IGF-I response analyses were performed for factors with significant impact on the somapacitan dose–IGF-I response (sex and oral oestrogen replacement, age, body weight and race). Of these, sex and oral oestrogen replacement were identified as the most influential factors.

It is well established that women, and in particular, women on oral oestrogen, respond less to GH treatment than men (4, 25), a difference that has largely been attributed to interactions between GH and sex steroids (25, 26). Indeed, oral oestrogen was reported to impair GH action and lower IGF-I levels (6, 25, 26, 27). The effect of oestrogen treatment is dependent on the route of administration, as transdermal oestrogen does not impair GH-mediated responses, likely due to a lower oestrogen exposure to the liver (25, 26, 27, 28, 29). In accordance with the above, the subgroup analyses indicated that the somapacitan dose–IGF-I response is lower in women, particularly women on oral oestrogen replacement, compared with men. The lower dose–response was the combined result of lower serum drug concentrations per somapacitan dose level and lower IGF-I responses at matching somapacitan concentrations, of which the dose–exposure effect appeared to be the most influential. The mechanism behind the reduced serum somapacitan levels in women (and women on oral oestrogen) may reflect an oestrogen-induced increase in the number of hepatic GH receptors (GHRs) (26, 30), potentially leading to increased GHR-mediated clearance of somapacitan and thereby decreased serum somapacitan concentrations. The reduced IGF-I response in these patients may be a result of the well-described effect of oestrogen as a stimulator of SOCS2 expression, a negative regulator of GHR signalling (26). The lower somapacitan dose–IGF-I response in women, and particularly women on oral oestrogen replacement, demonstrates why higher somapacitan doses are required in these patient groups compared with men. The current study thus extends the prior finding that higher doses of daily GH replacement are needed in women on oral oestrogen (5, 6, 29) to once-weekly somapacitan as well.

The subgroup analyses further indicated that patients aged ≥65 years need lower doses of somapacitan to reach normal range IGF-I SDS levels compared with younger patients. This is in line with observations from daily GH treatment, for which dose requirements are known to decline with age (2), indicating similar age-dependent dose–response effects for somapacitan and daily GH treatment. In addition to the age-dependent effect on dose–IGF-I responses, it has been observed that GH sensitivity and adverse effects increase with age (31). The lower GH doses in older patients may therefore also represent reduced tolerability. Furthermore, as GH secretion is known to decline with age in the general population (18), lower GH doses for replacement therapy in older patients are also in line with a reduced physiological GH requirement in non-GHD subjects.

Similar dose requirements to obtain normal range IGF-I SDS were predicted across the race groups, despite minor differences in dose–exposure and exposure–IGF-I responses. The similar responses observed for White/other and Asian Japanese subjects are in line with findings from phase 1 of the somapacitan development programme (32) as well as daily GH treatment (33), which showed similar PK and PD in Japanese and non-Asian subjects.

Subjects with obesity have been reported to obtain higher IGF-I responses per dose level of hGH (34, 35), possibly due to the interaction between GH and insulin on the hepatic GHR (36). The effect may also be linked to levels of growth hormone-binding protein (GHBP), as circulating GHBP levels are positively correlated with fat mass (37, 38, 39), as well as GH response in GH deficiency (39). Although the subgroup analyses also indicated a higher IGF-I response to serum somapacitan concentrations for patients with higher body weights, this was counteracted by lower exposures in these patients, resulting in similar dose requirements to obtain normal range IGF-I SDS levels across body weights, despite these significant weight-dependent effects. Further studies are needed to determine if somapacitan dose requirements remain stable during weight gain or weight loss for the individual patient.

Overall, the subgroup analyses extend the knowledge of GH sensitivity in patient populations and indicate that the patient demographics and characteristics affecting the dose–IGF-I response of once-weekly somapacitan are similar to those previously reported for daily GH treatment (2, 6).

## Conclusion

In conclusion, the clinical guidance on dosing of once-weekly somapacitan in patients with AGHD was supported by dose–exposure–IGF-I response analyses. Data from phase 3 supported the use of three starting dose groups in AGHD patients based on age and oral oestrogen therapy. The therapeutic dose range varied considerably across the AGHD population, indicating that the dose for each patient is best obtained with individual titration. For patients switching from daily GH treatment, the somapacitan maintenance dose (mg/week) was observed and predicted to be on average 8.2 (observed IQR of 6.7–9.1) times higher than the previous somatropin dose (mg/day). Higher starting doses for previously GH-treated patients, which may be used according to local regulations, were supported by modelling indicating that the IGF-I response generally remains within the normal range at these dose levels. Interpretations of missed dose simulations suggested that the weekly IGF-I profile of somapacitan allows for maintained IGF-I levels despite three days of delayed dosing compared with three missed somatropin doses. Finally, the characterisation of dose–exposure–IGF-I responses in patient subgroups provided insights on differences in dose requirements, in particular for females on oral oestrogen .

## Supplementary materials

Supplementary Material

## Declaration of interest

R J K, C H and M H R are employees of Novo Nordisk A/S and have equity interests in the company. B M K B has received occasional consulting honoraria from Aeterna Zentaris, Ascendis, Merck Serono and Novo Nordisk and has served as the PI of a research grant to Massachusetts General Hospital from Ascendis. G J has previously consulted for AstraZeneca, Merck, Serono, Novo Nordisk, Pfizer and Shire, has received lecture fees from Eli Lilly, Merck Serono, Novartis, Novo Nordisk, Pfizer, Otsuka and Shire and has received grant support from Novo Nordisk, Pfizer and Shire. Y T has received honoraria from Novo Nordisk, Novartis, Eli Lilly, Recordati Rare Disease, Otsuka Pharma and Ascendis Pharma and has received grant support from Ono Pharma, Teijin Pharma, Novo Nordisk, Kowa Pharma, Taisho Pharma, Daiichi Sankyo Pharma and Tanabe Mitsubishi Pharma.

## Funding

This work was supported by Novo Nordisk A/S, Denmark.
