# Supplementary material for: Dose–exposure–IGF-I response of once-weekly somapacitan in adults with GH deficiency
Source: Eur J Endocrinol. 2022 Apr 13;187(1):27–38. doi: 10.1530/EJE-21-1167 (PMC9175552; doi:10.1530/EJE-21-1167)
Supplement: Supplementary Material [file supplementary_material.pdf]

Dose–exposure–IGF-I response of once-weekly somapacitan

in adults with GH deficiency

Supplementary Data

Supplementary methods

Bioanalysis

Somapacitan plasma concentrations were measured using a validated somapacitan specific luminescent oxygen channelling immunoassay developed by Novo Nordisk. The lower limit of quantification (LLOQ) was 0.500 ng/mL. The assay was validated according to the current guidelines for bioanalytical method validation and performed with a precision (% coefficient of variation [CV]) of 3.59 to 12.4% (21.5% at LLOQ). The accuracy (% relative error [RE]) was -7.46 to 2.84% across the full assay range. Insulin-like growth factor I (IGF-I) was analysed using commercially available assay kits (Immuno Diagnostic Systems immunoassay [ISYS] assay). The precision (%CV) was determined to be in the range of 2.7–3.3%.

Data sets and data cleaning

Patients with at least one pharmacokinetic (PK) measurement were included in the PK data set, and patients with at least one PK and IGF-I measurement were included in the PK/pharmacodynamic (PD) data set. A total of 333 patients were randomised to somapacitan in the phase 3 trials (**Table 1**), and 330 were included in the analyses (**Table 2**). Two patients were not included as they had no PK samples, and one patient was excluded during data cleaning due to a missing dosing diary.

A total of 4470 PK samples and 5137 IGF-I samples were obtained. During data cleaning, 106 (2.4%) PK and 259 (5.0%) IGF-I records were excluded, the majority of which were excluded due to incomplete or ambiguous dosing records or collection of samples later than 168 hours (7 days) after planned sampling time. No imputation was applied for missing PK and IGF-I data.

Somapacitan population PK and PK/PD analysis methods

The phase 3 PK/PD models were based on previously published models developed using phase 1 data (1). The PK of somapacitan was described by a one-compartment model with dual first- and zero-order absorption through a transit compartment and with saturable elimination, and the PK/PD model was an indirect response model with an additive maximum IGF-I production rate (E_{max}) effect relationship between somapacitan PK and IGF-I rate of production. Key PK parameters were related to the relative bioavailability (F), correlating with the total exposure per mg somapacitan. Key PD parameters were the baseline IGF-I production rate (k_{in}), correlating with the observed baseline IGF-I levels, and E_{max}, correlating with the maximum effect predicted with somapacitan. The PK model was fitted to estimate structural parameters, residual variability and inter-individual variability (IIV) equivalent to F. The PK/PD model was fitted to estimate structural parameters, residual variability and IIV on k_{in} and E_{max}. Structural PK and PD parameters that were not identifiable with the sparse PK and IGF-I data sets obtained in phase 3 were fixed to those estimated with the phase 1 models based on full PK and IGF-I profiles (1).

Covariate models for somapacitan PK and PK/PD

Pre-defined covariates were tested in a stepwise manner for all parameters with IIV. Predefined covariates included body weight, sex (male, female without oral oestrogen, female with oral oestrogen), age group (>65, ≤65) and race (White/Other, Asian Japanese, Asian non-Japanese). Covariates significant at $p<0.01$ were retained in the final models (**Supplementary Table 2**). The reference profile was pre-specified as male, White, age <65 years and body weight of 85 kg. All pre-defined covariates significantly affected F and were included as covariates in the PK model. The following covariates significantly affected somapacitan PD parameters and were included in the PK/PD model: body weight, oral

oestrogen use and age on k_{in}, and body weight, sex, race (Asian non-Japanese) and age on E_{max}.

In addition to pre-defined covariates, the following exploratory covariates were tested: injection site (abdomen, thigh, unknown), growth hormone deficiency (GHD) onset (childhood, adulthood), age group (≤25 years, >25 years), body mass index (BMI) group (<25 kg/m², 25–35 kg/m², 35–40 kg/m² and >40 kg/m²) and diabetes status (yes, no). No exploratory covariates significantly affected PK or PD parameters of somapacitan.

All covariate values used in the models were baseline values. For the categorical covariate variables, at least 20 patients were required in each category. Race groups with an insufficient number of patients (Black or African American [n=4] and Hawaiian or Pacific Islander [n=1]) were pooled with the largest group (White) in the covariate analyses.

Evaluation of somapacitan and somatropin models

The final PK and PK/PD models were qualified by evaluation of the precision of fixed effect parameters, the shrinkage of random effects and the goodness-of-fit by visual inspection of model diagnostics plots, including prediction-corrected visual predictive checks (VPCs). VPCs are shown in **Supplementary Fig. 1** for the somapacitan PK model (**Supplementary Fig. 1A**), the somapacitan PK/PD model (**Supplementary Fig. 1B**) and the somatropin PK/PD model (**Supplementary Fig. 1C**). The models reproduced the overall trends of the phase 3 data, including the median and variability. Hence, the models provided accurate prediction of the observed data.

Sensitivity analyses showed similar results when dividing age groups at 60 years (>60, ≤60) and 65 years (>65, ≤65) in the PK and PK/PD models. Different age groups were used for starting dose groups (>60, ≤60) and for covariate age groups in the modelling analysis (>65, ≤65), due to a discrepancy between current clinical treatment guidelines (2) and the definition of geriatric populations in regulatory guidelines (3).

Dose–exposure-IGF-I estimation based on final somapacitan PK and PK/PD models

Estimates for the individual dose–exposure–IGF-I-response pairs at maintenance dose levels after titration were constructed based on individual estimates for PK and PK/PD parameters from the final PK and PK/PD models. Fixed dose levels were defined as the median of doses registered for an individual after the last titration visit. Average exposure (C_{avg}) and average IGF-I response ($IGF\text{-}I_{avg}$) were estimated from the area under the curve (AUC) of individual predicted profiles during maintenance treatment for the fixed dose level. No imputation was performed for subjects not completing titration (n = 6).

The individual dose–exposure–IGF-I-response curves across the somapacitan dose range of 0.1–8 mg were estimated from the AUC of individual predicted profiles using the final PK and PK/PD models.

Somatropin PK/PD analysis methods

The somatropin PK/PD model was based on data from three phase 3 trials in patients with adult growth hormone deficiency (AGHD) (**Tables 1** and **2**) (4-6). The PK of somatropin was described by a one-compartment model with linear absorption and clearance, while somatropin PK/PD was described using an indirect response model with additive E_{max} effect relationship between PK and IGF-I, like the PK/PD model for somapacitan. Somatropin model development and dose–response analyses were performed similarly to the procedures described for somapacitan.

Software

R version 3.2.3 or above (R Foundation for Statistical Computing, Vienna, Austria) was used for data file processing, explorative data analysis, exposure-response analyses, for plotting and simulation. NONMEM version 7.3 (ICON Development Solutions, Ellicott City, MD,

USA) and PsN (version 4.6.0) was used for the population PK and PK/PD analysis and simulation.

Supplementary Table 1: Ethics committees by country and trial

Country	Ethics committee	Trial
AU	1. Bellberry human Research Ethics committee	REAL 1
	2. Melbourne Health Human Research Ethics Committee	REAL 1
DE	1. Landesamt für Gesundheit und Soziales Ethik-Kommission des Landes Berlin	REAL 1, 2
DK	1. Regionshuset Viborg, De Videnskabsetiske Komiteer for Region Midtjylland	REAL 2
FR	1. CPP Ouest II, Maison de la Recherche Clinique	REAL 2
IL	1. Souraski Helsinki Committee	REAL 1
IN	1. Institutional Ethics Committee, KPC Medical College & Hospital	REAL 1
	2. Institutional Ethics Committee of Topiwala National Medical College and BYL Nair Charitable Hospital G	REAL 1
	3. Institutional Ethical Committee, Care Hospital, Care Convergence Centre, H.	REAL 1
	4. Ethics Committee, Apollo Hospitals	REAL 1
	5. Institutional Ethics Committee, Amrita Institute of Medical and Research Centre	REAL 1
JP	1. Hokkaido University Hospital IRB	REAL 1
	2. Tokyo Heart Center IRB	REAL 1
	3. Chiba University Hospital IRB	REAL 1, JP
	4. Toranomon Hospital IRB	REAL 1
	5. Nippon Medical School Hospital IRB	REAL 1, 2, JP
	6. Kitasato University Sagamihara IRB	REAL 1, 2, JP
	7. Kyoto Medical Center IRB	REAL 1, 2
	8. Central IRB of Clinical Research Network Fukuoka	REAL 1
	9. Yamagata University Hospital IRB	REAL 1, JP
	10. Okayama University Hospital IRB	REAL 1, JP
	11. Kagoshima University Hospital IRB	REAL 1, JP
	12. Teikyo University Hospital IRB	REAL 1, 2
	13. Yokohama Rosai Hospital IRB	REAL 1, JP
	14. IRB of Adachi Kyosai Hospital	REAL 1, JP
	15. Kobe University Hospital IRB	REAL 2, JP

	16.	IRB of Takahashi Clinic	REAL 2
	17.	Shimane University Hospital IRB	REAL 2
	18.	Institutional Review Board of National Hospital Organization Kyoto Medical Center	REAL JP
	19.	Fukuoka University Chikushi Hospital IRB	REAL JP
	20.	Osaka University Hospital IRB	REAL JP
LT	1.	Lithuanian Bioethics Committee	REAL 1
LV	1.	Ethics Committee for Clinical Research at Pauls Stradins Clinical University Hospital Development Society	REAL 1
MY	1.	Medical Research & Ethics Committee, National Institute of Health	REAL 1
PL	1.	Komisja Bioetyczna przy Okręgowej Izbie Lekarskiej w Krakowie	REAL 1
RO	1.	National Bioethics Committee for Medicines and Medical Devices	REAL 1
RU	1.	Ethics Committee at Ministry of Health of the Russian Federation	REAL 1
	2.	Local Ethics Committee of Federal State Budget Institution Endocrinology Research Centre	REAL 1
	3.	Ethics Committee of the State Budget Educational Institutional of Higher Professional Education “Siberian State Medical University” of the Ministry of Healthcare of Russian Federation	REAL 1
	4.	Ethics Committe of the State Budget Educational Institutional “Kazan State Medical Academy of the Ministry of Healthcare of the Russian Federation”. The Local Ethics Committee was changed on 20-Oct-2016 to: Local Ethics Committee of the Limited Liability Company of the Research Medical Complex “Your Health”	REAL 1
	5.	Ethics Committee within SBEI of HPE “Novosibirsk State Medical University of Ministry of Healthcare”	REAL 1
	6.	Ethics Committe of State Federal-Funded Educational Institution of Higher Professional Training I.M. Sechenov First Moscow State Medical University of the Ministry of Health of the Russian Federation	REAL 1
SE	1.	Regionala Etikprövningsnämnden i Göteborg	REAL 1, 2
TR	1.	Istanbul University Cerrahpaşa Medical Faculty Ethics Committee	REAL 1
UA	1.	The Ethic Committee State Institution “Institute of endocrinology and metabolism named after V.P. Komisarenko of the Academy of Medical Science of Ukraine”	REAL 1

UK	1. Helen Poole, NRES West Midlands	REAL 1, 2
	2. Dr Gillian Heap, The Research and Development Office, The Christie NHS Foundation Trust	REAL 1
	3. Martin Cooper, Research & Development Royal Devon and Exeter Hospital	REAL 1, 2
	4. Jamee Illingworth, Research & Development Department, Castle Hill Hospital	REAL 1
	5. Becky Chadwick, Research Development & Innovation, Department University Hospitals Coventry & Warwickshire NHS Trust	REAL 1
	6. Dr Nick McNally, Joint Research Office, UCLH NHS Foundation Trust	REAL 2
	7. Lifen Wang, The Research and Development Office, The Christie NHS Foundation Trust	REAL 2
	8. Mohammed Khan, Research & Innovation, Leeds Teaching Hospitals NHS Trust	REAL 2
	9. Dr Christopher Counsell, UHB Research Governance Office Education Centre	REAL 2
US	1. Partners Human Research Committee	REAL 1
	2. Columbia University Medical Center IRB	REAL 1
	3. Baylor College of Medicine IRB	REAL 1
	4. Copernicus Group IRB	REAL 1
	5. UCLA Office of the Human Research Protection Program	REAL 1
	6. Cleveland Clinic IRB	REAL 1
	7. University of Pennsylvania Office of Regulatory Affairs	REAL 1
	8. Washington University Human Research Protection Office	REAL 1
	9. VA Nebraska-Western Iowa Healthcare System Subcommittee of Human Studies of the Research and Development Committee	REAL 1
	10. Western Institutional Review Board	REAL 1
	11. Michigan State University Office of Regulatory Affairs Human Research Protection Program	REAL 1
	12. USC Health Sciences Campus Institutional Review Board	REAL 1
	13. OHSU Research Integrity	REAL 1
	14. UNMC Institutional Review Board	REAL 1
	15. Johns Hopkins Medicine Institutional Review Board	REAL 1
ZA	1. Wits Human Research Ethics Committee (WHREC)	REAL 1

Ethics committees are included for all trial sites that randomised and/or screened subjects in REAL 1, REAL 2 or REAL JP.

IRB, institutional review board.

Supplementary Table 2: Covariates included in the final PK and PK/PD models of somapacitan

PK parameter	Covariates included in the PK model	p-value	PK/PD parameter	Covariates included in the PK/PD model	p-value
**F**	Body weight	<0.0001	$\mathbf{k_{in}}$	Body weight	<0.0001
	Sex	<0.0001		Oral oestrogen intake	<0.0001
	Oral oestrogen intake	<0.0001		Age	0.0089
	Race (Asian Japanese)	<0.0001	$\mathbf{E_{max}}$	Body weight	0.0001
	Race (Asian non-Japanese)	<0.0001		Sex	0.0002
	Age	0.0006		Race (Asian non-Japanese)	0.0005
				Age	0.0013

Age groups (>65, ≤65). Race groups (White/Other, Asian Japanese, Asian non-Japanese), White/Other was set as reference. p-values were derived from the maximum likelihood difference between models with and without the covariate. Race effects on PK were tested as a covariate with two levels (Asian Japanese and Asian Non-Japanese versus White). No statistically significant difference was observed for White/Other versus Asian Japanese on E_{max}.

F, relative bioavailability; k_{in}, baseline IGF-I production rate; E_{max}, maximum IGF-I production rate; PD, pharmacodynamic; PK, pharmacokinetic.

Supplementary Table 3: Demographics and characteristics for starting dose groups

Category	Group	Patients ≤60 years	Patients >60 years	Females on oral oestrogen
**All**	N	188	70	72
**Gender (%)**	Female	37	36	100
	Male	63	64	0
**Race[a] (%)**	White/Other	63	53	72
	Asian Japanese	27	47	14
	Asian Non-Japanese	10	0	14
**Body weight (kg)**	Mean	77	78	67
**Age (years)**	Mean	43	67	36

Supplementary Table 4: Overview of dose adjustments in the titration period of the phase 3 trials

Titration group	Number of subjects (%)	Number of subjects needing 0 to 5 dose adjustments					
		0	1	2	3	4[a]	5[b]
**Titrating up**	186 (56%)	0	56 (17%)	53 (16%)	51 (15%)	26 (8%)	0
**Titrating down**	39 (12%)	0	29 (9%)	7 (2%)	3 (1%)	0	0
**Maintaining starting dose**	105 (32%)	103 (31%)	0	2 (1%)[c]	0	0	0
**Total**	330 (100%)	103 (31%)	85 (26%)	62 (19%)	54 (16%)	26 (8%)	0

Number of dose adjustments are included for the first titration period on somapacitan for each patient.

[a] Maximum number of dose adjustments allowed in REAL 1 and REAL 2.

[b] Maximum number of dose adjustments allowed in REAL JP.

[c] Two subjects titrated up or down but returned to starting dose level.

Supplementary Table 5: Predicted somapacitan and somatropin doses for similar mean IGF-I$_{avg}$

Mean IGF-I$_{avg}$ SDS	Somapacitan dose (mg/week)	Somatropin dose (mg/day)	Somapacitan (mg/week) vs somatropin (mg/day) ratio
0.0	2.0	0.25	8.1
0.6	3.0	0.37	8.2
1.1	4.0	0.49	8.2
1.5	5.0	0.61	8.2
1.9	6.0	0.74	8.1

Predicted somapacitan and somatropin doses for similar mean IGF-I$_{avg}$ SDS in the phase 3 somapacitan and somatropin populations, respectively.

Avg, average; IGF-I, insulin-like growth factor I; SDS standard deviation score.

Supplementary Fig. 1: Prediction-corrected visual predictive checks

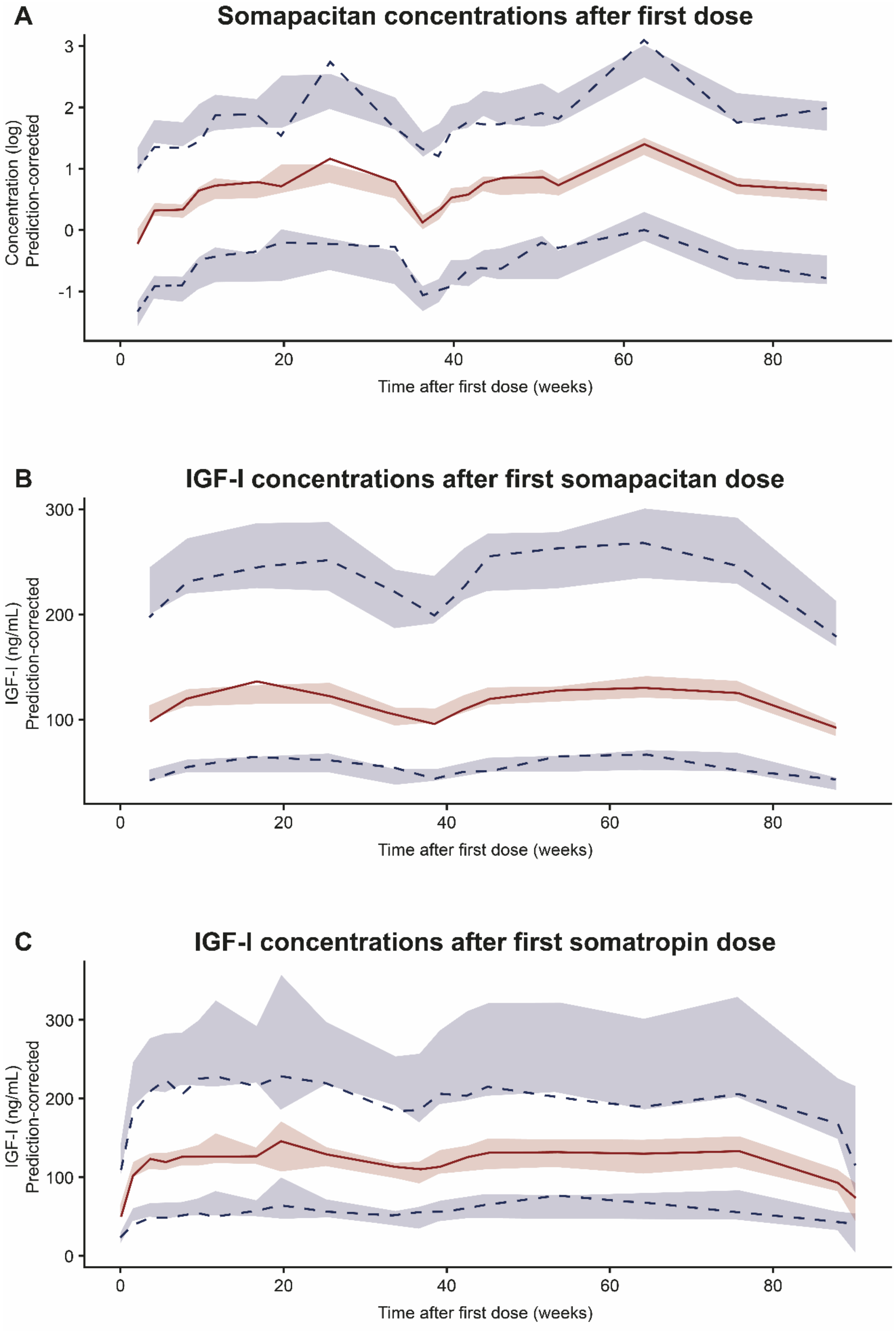

Data are prediction-corrected medians with 5th and 95th percentiles (red and blue lines) of somapacitan concentrations after first dose using the somapacitan PK model (a), IGF-I concentrations after first somapacitan dose using the somapacitan PK/PD model (b) or IGF-I concentrations after first somatropin dose using the somatropin PK/PD model (c). Shaded areas are 95% confidence intervals around the prediction corrected medians and 5th and 95th percentiles based on 1000 simulations with the somapacitan and somatropin models. IGF-I, insulin-like growth factor I; PD, pharmacodynamic; PK, pharmacokinetic.

References

1. Juul RV, Rasmussen MH, Agersø H, Overgaard RV. Pharmacokinetics and pharmacodynamics of once-weekly somapacitan in children and adults: Supporting dosing rationales with a model-based analysis of three phase I trials. Clin Pharmacokinet. 2018;58 (1):63-75.

2. Yuen KCJ, Biller BMK, Radovick S, Carmichael JD, Jasim S, Pantalone KM, Hoffman AR. American Association of Clinical Endocrinologists and American College of Endocrinology guidelines for management of growth hormone deficiency in adults and patients transitioning from pediatric to adult care. Endocr Pract. 2019;25 (11):1191-232.

3. CPMP/ICH/379/95 - Note for guidance on studies in support of special populations: Geriatrics. Canary Wharf, London: EMA. Published March 1994.

4. Johannsson G, Gordon MB, Rasmussen MH, Holme Håkonsson I, Karges W, Sværke C, Tahara S, Takano K, Biller BMK. Once-weekly somapacitan is effective and well tolerated in adults with GH deficiency: A randomized phase 3 trial. J Clin Endocr Metab. 2020;105 (4):e1358-e76.

5. Johannsson G, Feldt-Rasmussen U, Håkonsson IH, Biering H, Rodien P, Tahara S, Toogood A, Rasmussen MH, Group RS. Safety and convenience of once-weekly somapacitan in adult GH deficiency: A 26-week randomized, controlled trial. Eur J Endocrinol. 2018;178 (5):491-9.

6. Otsuka F, Takahashi Y, Tahara S, Ogawa Y, Højby Rasmussen M, Takano K. Similar safety and efficacy in previously treated adults with growth hormone deficiency randomized to once-weekly somapacitan or daily growth hormone. Clin Endocrinol (Oxf). 2020;93 (5):620-8.
